# Identification of novel proteins and mechanistic pathways associated with early-onset hypertension by deep proteomic mapping of resistance arteries

**DOI:** 10.1016/j.jbc.2021.101512

**Published:** 2021-12-18

**Authors:** Joakim A. Bastrup, Christian Aalkjær, Thomas A. Jepps

**Affiliations:** 1 Vascular Biology Group, Department of Biomedical Sciences, University of Copenhagen, Copenhagen, Denmark; 2 Department of Biomedicine, Aarhus University, Aarhus, Denmark

**Keywords:** hypertension, vascular, arteries, proteomics, extracellular matrix, remodeling, BP, blood pressure, DIA-MS, data-independent acquisition–mass spectrometry, ECM, extracellular matrix, GO, Gene Ontology, KEGG, Kyoto Encyclopedia of Genes and Genomes, MS, mass spectrometry, PCA, principal component analysis, ROS, reactive oxygen species, SHR, spontaneously hypertensive rat, VSMC, vascular smooth muscle cell, WKY, Wistar Kyoto

## Abstract

Resistance arteries are small blood vessels that create resistance to blood flow. In hypertension, resistance arteries undergo remodeling, affecting their ability to contract and relax appropriately. To date, no study has mapped the hypertension-related proteomic changes in resistance arteries. Using a novel data-independent acquisition–mass spectrometry (DIA-MS) approach, we determined the proteomic changes in small mesenteric and renal arteries in pre- and early-onset hypertension from the spontaneously hypertensive rat (SHR) model, which represents human primary hypertension. Compared with normotensive controls, mesenteric arteries from 12-week-old SHRs had 286 proteins that were significantly up- or downregulated, whereas 52 proteins were identified as up- or downregulated in mesenteric arteries from 6-week-old SHRs. Of these proteins, 18 were also similarly regulated in SHR renal arteries. Our pathway analyses reveal several novel pathways in the pathogenesis of hypertension. Finally, using a matrisome database, we identified 38 altered extracellular-matrix-associated proteins, many of which have never previously been associated with hypertension. Taken together, this study reveals novel proteins and mechanisms that are associated with early-onset hypertension, thereby providing novel insights into disease progression.

Hypertension is the main risk factor for cardiovascular diseases and is a major global health burden, with increasing prevalence (1). Although many studies have investigated specific genes, proteins, and pathways that are altered in arteries from hypertensive animals and humans, there is no overview of the changes that occur in arteries during hypertension. As such, the pathophysiology of essential hypertension remains unclear. To advance research in the field of hypertension, we need a better overview of the changes occurring in arteries, which will promote new research ideas and potential therapeutic targets (2).

In hypertension, resistance arteries undergo eutrophic and/or hypertrophic remodeling, which contributes to increased peripheral resistance (3). In patients with essential hypertension and the spontaneously hypertensive rat (SHR), inward eutrophic remodeling predominates (3). Several mechanisms are proposed to influence vascular remodeling in hypertension (4), including apoptosis (5), expanded extracellular matrix (ECM) (6), vascular inflammation (7), and dysfunctional endothelium (8). In addition, several contractile and dilatory mechanisms are compromised in arteries from hypertensive animals and humans, which also contribute to the development and persistence of hypertension. These maladaptive changes in the vessel wall influence the development and cardiovascular complications of hypertension. Although proteins have been implicated in vascular remodeling in hypertension, these proteins do not work in isolation.

Mass spectrometry (MS) analysis has advanced rapidly over the past 2 decades and demonstrated clear advantages in mapping complex biological systems with high reproducibility (9, 10). Previously, MS analysis identified proteomic changes in the kidney (11), aortic smooth muscle (12), and left ventricular myocardium (13) in the SHR. The SHR develops elevated blood pressure (BP) between 7 and 15 weeks of age and mimics the central phenotypic changes observed in human essential hypertension, such as cardiac hypertrophy and vascular remodeling (14). To date, no study has mapped the proteomic changes in the resistance arteries of the SHR or in patients with essential hypertension.

The aim of this study was to investigate protein changes and mechanistic pathways in mesenteric resistance and renal arteries from the SHR, which is an ideal model for studying human essential hypertension without confounding lifestyle and environmental factors. Contrary to previous proteomic studies, we utilized next-generation data-independent analysis (DIA)-MS to achieve deep proteomic coverage of resistance arteries allowing us to identify novel proteins and map the pathophysiological mechanisms contributing to vascular remodeling and early-onset hypertension.

## Results

### Study overview and workflow

**Figure 1:**
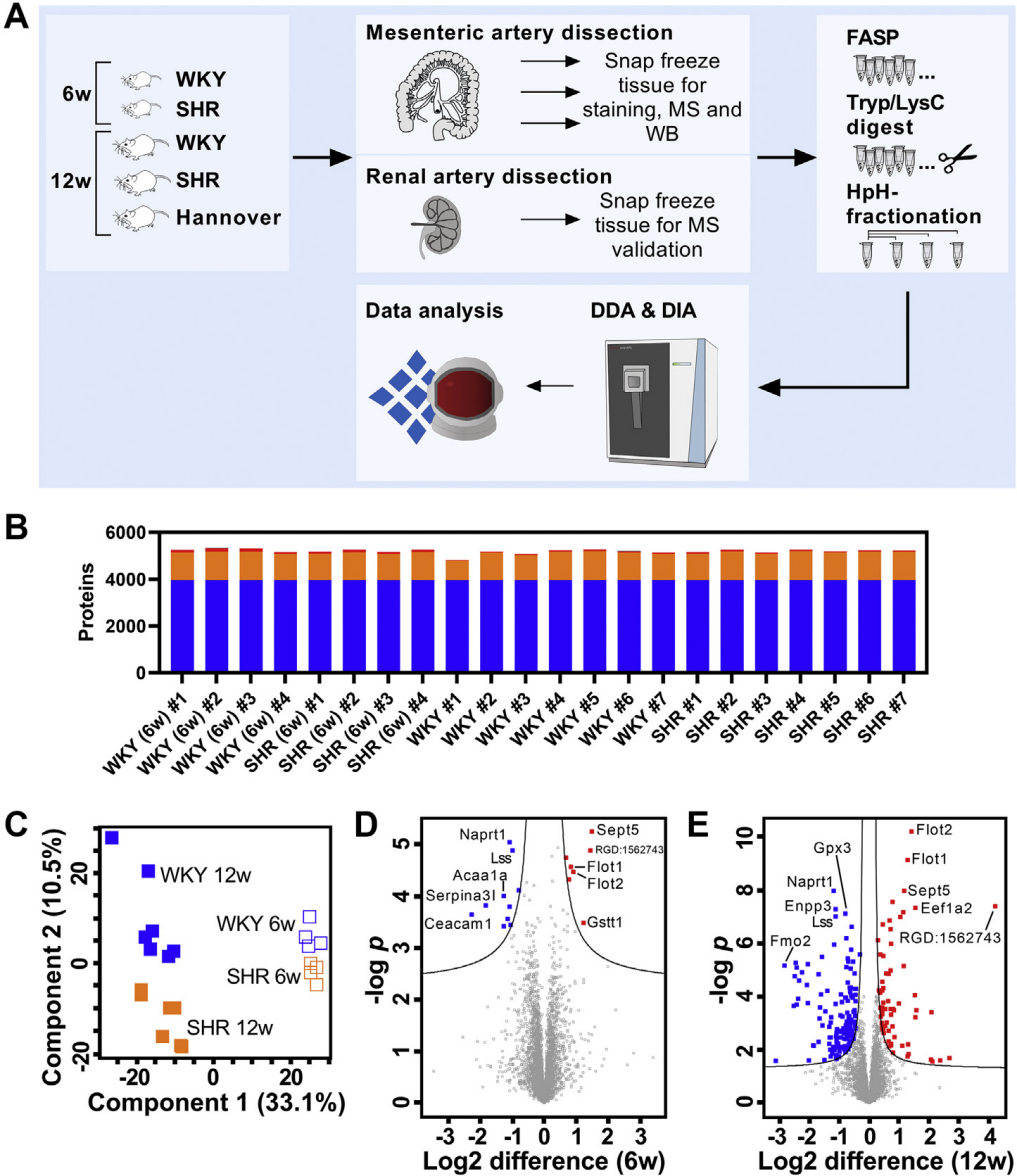
**Study overview and presentation of proteomic data.***A*, methodological overview of the proteomic analysis in this study. Small mesenteric arteries and renal arteries were extracted from two cohorts of Wistar Kyoto (WKY) and spontaneously hypertension rat (SHR) at 6 weeks (n = 4) and 12 weeks of age (n = 7), respectively. An additional cohort of 13-week-old Wistar Hannover (Hannover, n = 6) rats was included for additional validation. Extracted mesenteric artery tissue was snap frozen and analyzed by histological staining, mass spectrometry (MS) or Western blot (WB). Renal artery tissue was analyzed by MS analysis as additional validation. Samples for MS analysis were prepared by filter-aided sample preparation (FASP) protocol with LysC and trypsin for protein digestion. High pH (HpH)-fractionation was applied on two pooled samples containing mesenteric arteries from SHR or WKY, respectively. Samples were analyzed by liquid chromatography–tandem MS (LC-MS/MS) using data-independent (DIA) and data-dependent acquisition (DDA) followed by data analysis using Spectronaut (Biognosys AG). *B*, stacked bar representation of proteins identified by DIA MS across mesenteric artery samples (*blue* = complete identifications, *orange* = shared in 50% of runs, *red* = sparse identifications). Unique identifications are not visible on this graph. *C*, principal component analysis (PCA) plot of log2 transformed label-free quantification (LFQ) intensities associated with samples (*blue squares* = WKY controls, *orange squares* = SHRs, *filled squares* = 12-week old). Components 1 and 2 are presented. *D* and *E*, volcano plots comparing protein abundance in 6-week (*D*) and 12-week (*E*) old mesenteric artery samples from SHR and WKY controls.

We investigated the protein composition of mesenteric artery samples from the SHR by label-free DIA quantification. Initially, we analyzed freshly isolated small mesenteric arteries from SHR and Wistar Kyoto (WKY) at both 6 and 12 weeks of age (Fig. 1*A*). BP in the SHR begins to increase at ∼6 weeks of age, leading to a chronic elevated BP from ∼12 weeks (14). Our study was designed to capture the critical changes that occur in the arterial wall during the early-onset of high BP; thus, these time points were selected to represent prehypertensive and early-onset hypertensive phenotypes and avoid confounding pathological changes associated with long-term chronic hypertension.

To achieve deep proteomic coverage, we generated a hybrid DIA library that was based on both high pH (HpH) reversed-phase peptide fractionated mesenteric artery samples and the strength of direct DIA to maximize the protein identification (15). Our hybrid library contained a total of 7450 proteins (73,378 peptides; 106,796 precursors; Fig. S1). Taking advantage of our hybrid DIA library, we identified a total of 4725 proteins in mesenteric artery samples from both SHR and WKY. Of these, 3956 proteins were consistently observed across all samples, suggesting high proteomic overlap and reproducibility between the mesenteric artery samples from SHRs and WKY controls (Fig. 1*B*).

Unbiased principal component analysis (PCA) revealed distinct clusters of mesenteric artery samples corresponding to age (6 and 12 weeks) along component 1 and phenotype (WKY and SHR) along component 2 (Fig. 1*C*). The separation of 6-week-old SHR and WKY control samples on component 2 was less compared with the 12-week-old samples, confirming higher proteomic similarity between the 6-week-old phenotypes. Using a volcano plot, we identified 17 regulated proteins, which accounted for the segregation of 6-week-old SHR and WKY control samples, including proteins such as Sept5, RGD:1562743, Flot1, Flot2, Gstt1, Naprt1, Lss, Acaa1a, Serpina3l, and Ceacam1 (Fig. 1*D*). A total of 212 regulated proteins were identified in a volcano plot analysis when comparing 12-week-old SHR and WKY controls and supported the clear segregation seen in the PCA plot (Fig. 1, *C* and *E*). Almost all of the regulated proteins identified in the 6-week-old comparison (15/17) were shared across the two time point comparisons (Fig. 1, *D* and *E*).

### Identification of 286 significantly regulated proteins in 12-week-old SHR mesenteric arteries compared with normotensive controls

**Figure 2:**
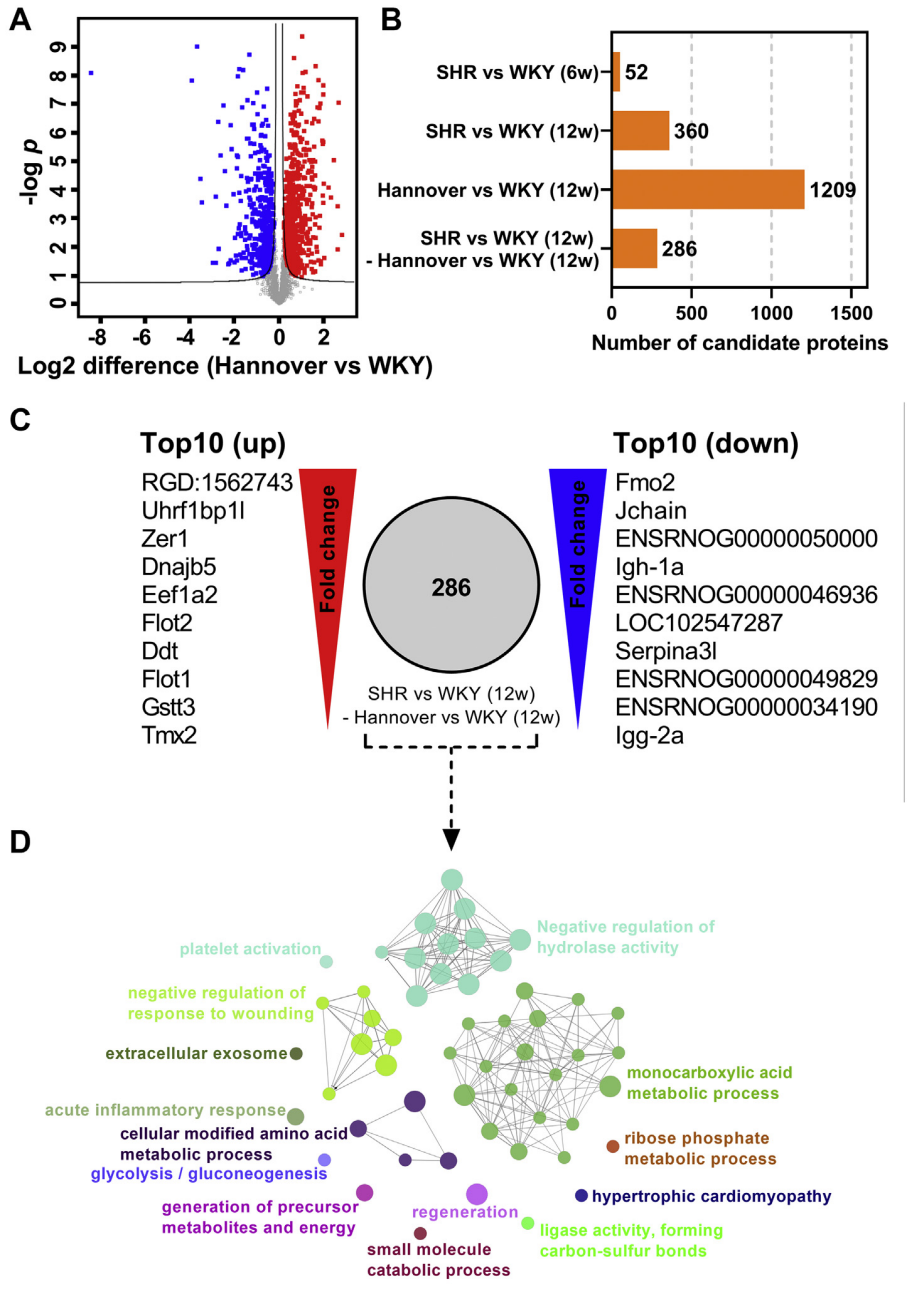
**Proteomic changes in hypertensive rat model.***A*, volcano plot comparing protein abundance in mesenteric artery samples from outbred Hannover and inbred Wistar Kyoto (WKY) strains. *B*, bar representation depicting number of significantly regulated proteins identified when comparing WKY, spontaneous hypertension rat model (SHR), and Hannover control at different ages using Student *t* test comparison. *C*, representation of top ten up- (*left*) and top ten downregulated (*right*) proteins from previous comparison (SHR *versus* Wistar Kyoto (WKY) (12-week-old (12w)) minus Hannover *versus* WKY (12w)). *D*, ClueGO-enriched network of significantly regulated proteins (n = 286). The size of each node is based on the enrichment significance score. Each cluster of related terms is labeled with the highest significance score of annotated Gene Ontology (GO) or Kyoto Encyclopedia of Genes and Genomes (KEGG) terms after *p*-value correction. Nodes are linked by the kappa score level (≥0.4).

The SHR is derived from the WKY (14) and inbred to perpetuate the hypertensive phenotype. Unfortunately, the litter-matched WKYs, from which the SHRs were identified, were not kept for inbreeding as a control. Subsequent attempts have been made to inbreed WKYs as a control, which is not ideal, thus the differences between the SHR and WKY controls might be due to strain differences and genetic drift rather than strain difference in BP (16). To control for this limiting factor in the animal model, we performed an additional DIA-MS analysis of mesenteric artery samples from six 13-week-old outbred Wistar Hannover rats. When comparing Hannover to WKY control, we observed a pronounced difference in the volcano plot (Fig. 2*A*). This difference was supported by a Student *t* test comparison identifying 1209 significantly regulated proteins between the normotensive Wistar strains (Fig. 2*B*).

The inclusion of SHR and WKY controls at two time points allowed analyses of relative proteomic differences in prehypertensive (6 weeks) and early-onset hypertensive (12 weeks) stages. Using Student *t* test analysis, we identified 52 and 360 significantly regulated proteins when comparing the mesenteric arteries of the SHRs to the WKY controls at 6 and 12 weeks of age, respectively (Fig. 2*B*; Tables S1 and S2). All candidate proteins contained between 2 and 307 unique peptides and were filtered by *p* and *q* values <0.05.

We compared the 360 significantly regulated proteins with those identified in the Hannover *versus* WKY control list and removed overlapping proteins that changed in the same direction. This resulted in the removal of 74 proteins from the SHR *versus* WKY list as these could be attributed to strain differences or genetic drift in the WKY rather than strain difference in BP. This conservative approach left us with 286 significantly regulated proteins (Fig. 2*B*; Tables S3 and S4). We focused on these proteins in the subsequent analysis to limit confounding changes caused by stain difference and genetic drift.

### Fourteen different biological processes are associated with the protein changes in mesenteric arteries from the SHR

We ranked the top ten upregulated and downregulated candidate proteins based on the log2-transformed differences (Fig. 2*C* and Table 1). Examples included immunoglobulins, such as RGD:1562743 (Igkc), Igh-1a and Igg-2a, and thioredoxin-associated proteins, including Gstt3 and Tmx2. These proteins are highly associated with immune mechanisms, and the changes in expression levels suggested involvement of the immune system in SHRs.

**Table 1:** Top ten regulated proteins in mesenteric arteries

Protein group(s)	Genes	Protein description	Log2 difference	*p*-value	Unique peptides
Top ten upregulated proteins					
P01836	RGD:1562743	Ig kappa chain C region, A allele	4.24	4.08E-08	7
M0R8V0	Uhrf1bp1l	Chorein_N domain-containing protein	2.10	3.87E-04	4
F1LQI6	Zer1	Zyg-11-related, cell cycle regulator	1.60	3.33E-04	4
A0A0G2K9H9;D3ZB76	Dnajb5	J domain-containing protein;Isoform of A0A0G2K9H9, J domain-containing protein	1.56	5.97E-04	5
P62632	Eef1a2	Elongation factor 1-alpha 2	1.55	4.53E-08	12
Q9Z2S9	Flot2	Flotillin-2	1.42	6.19E-11	55
P80254	Ddt	D-dopachrome decarboxylase	1.31	6.35E-03	6
Q9Z1E1	Flot1	Flotillin-1	1.30	7.34E-10	54
D3Z8I7	Gstt3	GST N-terminal domain-containing protein	1.17	7.15E-06	9
Q5XIK2	Tmx2	Thioredoxin-related transmembrane protein 2	1.11	7.10E-03	7
Top ten downregulated proteins					
G3V6F6	Fmo2	Isoform of Q6IRI9, Dimethylaniline monooxygenase [N-oxide-forming]	−2.85	7.05E-06	29
G3V6G1	Jchain	Immunoglobulin joining chain	−2.53	2.28E-04	7
P20767	ENSRNOG00000050000	Ig lambda-2 chain C region	−2.50	1.72E-05	6
P20761	Igh-1a	Ig gamma-2B chain C region	−2.46	5.46E-06	19
F1LVL4	ENSRNOG00000046936	Ig-like domain-containing protein	−2.43	1.98E-04	3
F1LYK4	LOC102547287	Zinc finger protein 728-like	−2.39	1.19E-04	2
P05544	Serpina3l	Serine protease inhibitor A3L	−2.35	2.54E-05	46
F1LTN6	ENSRNOG00000049829	Uncharacterized protein	−2.08	3.75E-05	20
A0A0G2K477	ENSRNOG00000034190	Isoform of F1LPW0, Immunoglobulin heavy constant mu	−2.06	6.22E-06	12
P20760	Igg-2a	Ig gamma-2A chain C region	−2.05	1.76E-04	25

Description of the top ten up- and downregulated proteins identified when comparing the proteomic profile of 12-week-old mesenteric arteries from spontaneously hypertensive rats (SHR) and normotensive Wistar Kyoto rats (WKY). The number of unique peptides (precursors) identified per protein is included.

The top ten regulated proteins had in average 16 unique peptides per protein, which suggests strong identification and gives confidence in the data (Table 1). To determine whether protein expression in any particular pathway was affected in the SHR, we performed pathway analysis on the entire list of significantly regulated proteins (=286) using ClueGO (17) (Fig. 2*D* and Table S5). The analysis identified 14 clusters of related Gene Ontology (GO) and Kyoto Encyclopedia of Genes and Genomes (KEGG) terms (Fig. 2*D* and Table 2). Each node was represented by 3 to 36 proteins and had an enrichment significance score between 4.85E-02 and 5.44E-10 using the Bonferroni step-down method (Table 2).

**Table 2:** Pathway analysis of proteins associated with hypertension phenotype

Pathway	Ontology source	Corrected *p*-value	Upregulated	Downregulated
Acute inflammatory response	GO Biological Process	3.41E-03	Park7	Ahsg, Apoa2, F2, Icam1, Itih4, Kng1, Kng2, Pla2g4a, Serpinb9
Cellular modified amino acid metabolic process	GO Biological Process	1.13E-04	Aldh7a1, Ckb, Gstt1, Gstt3, Park7, Tmlhe	Aldh9a1, Cpq, Crot, Gstm5, Idh1, Kyat1, Por, Slc27a1
Extracellular exosome	GO Cellular Component	2.70E-02	Hspd1, Park7, Pdcd6ip, Sdcbp	Alb, Icam1, Sri
Generation of precursor metabolites and energy	GO Biological Process	5.07E-04	Agl, Aldoc, Atp5po, Coq9, Cyc1, Ndufa5, Oxct1, Park7, Ppp1cc, Prkag2	Adh1, Apoc3, Crot, Cyb5a, Eno2, Idh1, Pgam2, Pgd, Por, Tkt
Glycolysis/Gluconeogenesis	KEGG	9.96E-03	Acss1, Aldh7a1, Aldoc	Adh1, Aldh9a1, Eno2, Pgam2
Hypertrophic cardiomyopathy	KEGG	4.28E-02	Atp2a2, Itga7, Prkag2, Ryr2	Agt, Itga9, Sgca
Ligase activity, forming carbon-sulfur bonds	GO Molecular Function	3.00E-02	Acss1, Uba3	Acsl5, Slc27a1, Slc27a4
Monocarboxylic acid metabolic process	GO Biological Process	1.76E-05	Acss1, Aldoc, Mapk14, Park7, Prkag2	Acaa1a, Acsl5, Adh1, Agt, Apoa4, Apoc1, Apoc3, Ces1d, Crot, Eno2, Ephx1, Hyi, Idh1, Kyat1, Pgam2, Pgd, Pla2g4a, Por, Sgpl1, Slc27a1, Slc27a4, Tecr
Negative regulation of hydrolase activity	GO Biological Process	5.44E-10	Cnn3, Crim1, Ddx3x, Farp1, Fkbp1a, Gas6, Park7, Pcsk1n, Ppp1r14a, Rock1	Agt, Ahsg, Apoa2, Apoc1, Apoc3, Itih2, Itih3, Itih4, Kng1, Kng2, LOC297568, LOC299282, Mug2, Por, Serpina3c, Serpina6, Serpinb9, Serpind1, Slc27a4, Sort1
Negative regulation of response to wounding	GO Biological Process	8.85E-05	Cask, Neo1, Phldb2	Cers2, Cpb2, F2, Kng1, Kng2, Proc, Thbd
Platelet activation	KEGG	1.36E-02	Gucy1a2, Itpr1, Mapk14, Mylk, Ppp1cc, Rock1	Col3a1, F2, Pla2g4a
Regeneration	GO Biological Process	4.72E-04	Aldoc, Gas6, Itpr1, Lamb2, Mapk14, Mustn1, Neo1, Ptgfrn	Adh1, Ahsg, Apoa2, Apoa4, Cers2, Cpb2, Cpq, Grn, Sgca
Ribose phosphate metabolic process	GO Biological Process	1.67E-02	Acss1, Aldoc, Atp5po, Ctps1, Cyc1, Gucy1a2, Prkag2, Uprt	Acsl5, Coasy, Crot, Eno2, Mcee, Pde4d, Pgam2, Tkt
Small molecule catabolic process	GO Biological Process	4.28E-02	Esd, Gnpda2, Oxct1, Park7	Acaa1a, Adh1, Ces1d, Crot, Ddah1, Eno2, Gpd2, Kyat1, Pgd, Slc27a4

ClueGO-enrichment analysis of significantly regulated proteins identified in mesenteric arteries from comparing 12-week-old Wistar Kyoto (WKY) and spontaneously hypertensive rats (SHR). The annotations represent related Gene Ontology (GO) or Kyoto Encyclopedia of Genes and Genomes (KEGG) terms enriched as nodes. Each node is labeled with the term having the highest significance. *p*-value correction was calculated by Bonferroni step-down method.

### Mapping of the extracellular-matrix-associated protein changes in the SHR mesenteric arteries

**Figure 3:**
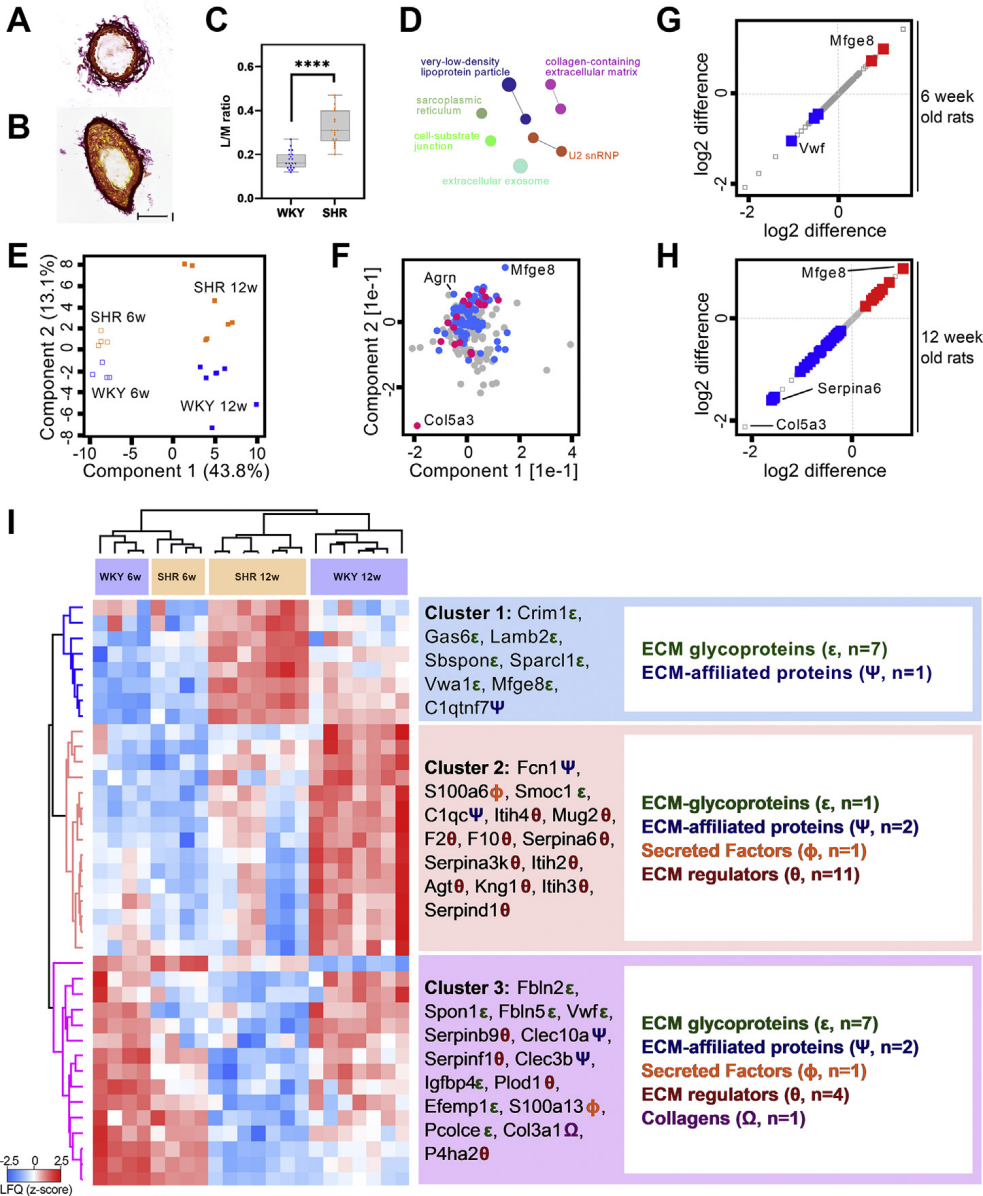
**Regulation of the matrisome in hypertensive rat model.** Sirius red staining of small mesenteric arteries in 12-week-old (*A*) Wistar Kyoto (WKY) and (*B*) spontaneous hypertension rat (SHR) model (20× lens; scale bar = 100 μm). *C*, media-to-lumen (M/L) ratio count in mesenteric arteries from the WKY and SHR (box plot representation; whiskers shows minimum and maximum values; student *t* test comparing WKY and SHR; WKY: n = 20 cross sections in two rats; SHR: n = 19 cross sections in two rats; ∗∗∗∗ *p* < 0.0001). *D*, ClueGO-enriched network of significantly regulated proteins described in Figure 2*B*. The protein list was enriched against the Gene Ontology (GO) cellular component database. *E*, principal component analysis (PCA) plot of log2 transformed label-free quantification (LFQ) intensities associated with samples (*blue squares* = WKY controls, *orange squares* = SHRs, *filled squares* = 12-week-old). Components 1 and 2 are presented. *F*, protein loadings of the PCA plot shown in (*E*). *G* and *H*, scatter plot showing protein abundance of matrisome-associated proteins from the total protein list. Statistically significant proteins from 6- and 12-week-old WKY control and SHR comparisons are shown in *red* (upregulated) or *blue* (downregulated) *squares*. *I*, unsupervised hierarchical clustering of significant proteins in (*H*). Z-scored LFQ values are depicted. ε, ECM-glycoproteins; Ψ, ECM-affiliated proteins; φ, Secreted factors; θ, ECM regulators; Ω, collagens.

Arteries from the SHR undergo vascular remodeling with thickening of the wall accompanied by reduction in lumen diameter (3). Using Sirius red staining, we confirmed the presence of vascular remodeling in 12-week-old SHRs compared with WKY controls (Fig. 3, *A* and *B*). In a blinded image analysis, we observed significant increases in media-to-lumen ratio in mesenteric arteries from the SHR compared with WKY controls, validating the presence of hypertrophic remodeling (Fig. 3*C*; Student *t* test comparison, *p* < 0.0001, t = 7.329, df = 37). Remodeling of the ECM has been associated with vascular remodeling (6). To explore this, we took advantage of our list of significantly regulated proteins and enriched for cellular components in an additional pathway analysis using ClueGO, which identified the collagen-containing extracellular matrix pathway (Fig. 3*D* and Table S6). To further elucidate this association, we compared our total protein list (before removal of the outbred Hannover-associated proteins) to a “matrisome” gene list containing ECM and ECM-associated proteins (18, 19) and identified a total of 228 proteins that were associated with the matrisome. A new unbiased PCA plot, based on the matrisome-associated proteins only, revealed an almost identical clustering of samples as observed in Figure 1*C* that included all proteins (Figs. 1*C* and 3*E*). Notably, collagen Col5a3 and the two ECM glycoproteins, Agrn and Mfge8, showed specific enrichment toward WKY controls and SHRs, respectively (Fig. 3*F*). Only six proteins (Mfge8, Plod1, F9, Vwf, P4ha2, and Col5a3) were significantly regulated in the 6-week comparison (Fig. 3*G*), whereas 38 proteins were regulated in the 12-week comparison (Fig. 3*H*). Mfge8 was identified as the most upregulated ECM protein in both 6- and 12-week-old rats (*p* = 5.00E-04 and 2.00E-04, respectively). Vwf was the most downregulated protein at 6 weeks (*p* = 3.60E-04, Fig. 3*G*) while Serpina6 was the most downregulated protein at 12 weeks (*p* = 8.28E-05; Fig. 3*H*). Although it did not reach statistical significance in the 12-week comparison, Col5a3 was the most downregulated protein identified with a difference of −2.14 (*p* = 0.073; Fig. 3*H*).

Unsupervised hierarchical clustering of significantly regulated ECM-associated proteins revealed three major groups (Fig. 3*I*). Cluster 1 was mainly upregulated in 12-week-old SHRs and contained mostly ECM glycoproteins (ε, n = 7/8; Fig. 3*I*). Cluster 2 was particularly upregulated in 12-week-old WKY controls and contained mostly ECM regulators (θ, n = 11/15; Fig. 3*I*). Cluster 3 contained a mixture of several ECM types and was generally upregulated in both 6- and 12-week-old WKY controls (Fig. 3*I*).

### Maintained protein changes across vascular beds of the 12-week-old SHR: Analysis of the renal arteries

**Figure 4:**
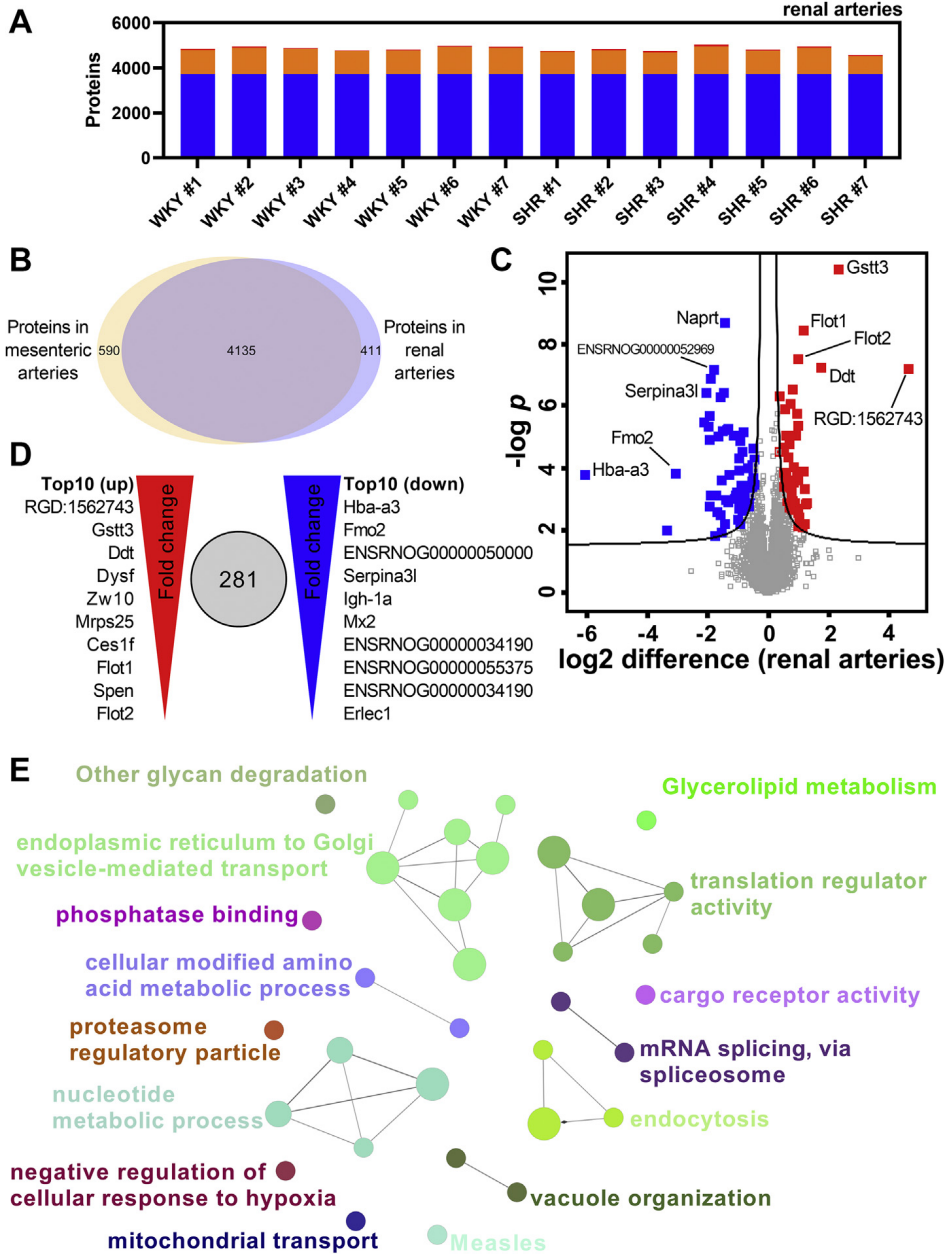
**Pathway analysis of proteins associated with early-onset hypertension in renal arteries from the SHR.***A*, stacked bar representation of protein groups identified by data-independent acquisition mass spectrometry (DIA MS) across renal artery samples (*blue* = complete identifications, *orange* = shared in 50% of runs, *red* = sparse identifications). *B*, Venn diagram showing total number of exclusive and shared protein groups identified in mesenteric and renal arteries (*orange* and *blue circle*, respectively). *C*, volcano plot comparing protein abundance in 12-week-old renal artery samples from spontaneous hypertensive rat (SHR, n = 7) and Wistar Kyoto (WKY, n = 7) control. *D*, representation of top ten up- (*left*, *red*) and top ten downregulated (*right*, *blue*) proteins when comparing 12-week-old SHR and WKY control. *E*, ClueGO-enriched network of significantly regulated proteins identified when comparing 12-week-old SHRs and WKY controls with *t* test analysis. The protein list was enriched against the Gene Ontology (GO) and Kyoto Encyclopedia of Genes and Genomes (KEGG) databases.

To determine which proteomic changes of early-onset hypertension that were identified in the mesenteric arteries and were maintained in a different vascular bed, we analyzed renal arteries from the SHR. Using the same sample preparation and DIA-MS setup, we consistently observed 3727 proteins across renal artery samples that were shared between SHRs and WKY controls at both 6 and 12 weeks of age (Fig. 4*A*). A total of 4546 different proteins were identified across all renal artery samples, which was similar to the number identified in mesenteric artery samples (4725). Furthermore, the two artery types shared 4135 proteins, suggesting a high proteomic overlap between the renal and mesenteric arteries (Fig. 4*B*). Conversely, we also observed 590 and 411 proteins that were observed exclusively in the mesenteric and renal arteries, respectively (Fig. 4*B*).

When comparing renal artery samples from 12-week-old SHRs with WKY controls, we observed pronounced differences in a volcano plot (Fig. 4*C*). An unpaired *t* test comparison identified 281 significantly regulated proteins between the 12-week-old WKY and SHR renal arteries. Of these, 57 were shared with the significantly regulated protein list identified in the 12-week-old mesenteric artery comparison. The majority of the top regulated proteins were similarly regulated in mesenteric arteries and suggested a preserved regulation across vascular beds (Fig. 4, *C* and *D*; Tables 1 and 3). Furthermore, the top ten regulated proteins detected in the renal arteries had in average 20 unique peptides per protein, which suggests strong identification and gives confidence in the data (Table 3).

**Table 3:** Top ten regulated proteins in renal arteries

Protein group(s)	Genes	Protein description	Log2 difference	*p*-value	Unique peptides
Top ten upregulated proteins					
P01836	RGD:1562743	Ig kappa chain C region, A allele	4.64	5.96E-08	7
D3Z8I7	Gstt3	GST N-terminal domain-containing protein	2.33	3.84E-11	9
P80254	Ddt	D-dopachrome decarboxylase	1.73	5.47E-08	11
A0A0G2K7B6	Dysf	Dysferlin	1.26	1.33E-03	68
Q4V8C2	Zw10	Centromere/kinetochore protein zw10 homolog	1.26	1.43E-03	10
Q4QR80	Mrps25	28S ribosomal protein S25, mitochondrial	1.19	4.41E-04	6
F1LLV6;M0R7R1;Q63010	Ces1f;Ces1f;-	Isoform of P10959, Carboxylic ester hydrolase; Isoform of P10959, Carboxylic ester hydrolase; Liver carboxylesterase B-1	1.16	1.21E-04	8
Q9Z1E1	Flot1	Flotillin-1	1.16	3.57E-09	40
F1M455;F1M816	Spen	Spen family transcriptional repressor; Isoform of F1M455, RCG30673	1.02	3.70E-03	2
Q9Z2S9	Flot2	Flotillin-2	1.00	3.03E-08	44
Top ten downregulated proteins					
Q63910	Hba-a3	GLOBIN domain-containing protein	−6.06	1.64E-04	17
G3V6F6	Fmo2	Isoform of Q6IRI9, Dimethylaniline monooxygenase [N-oxide-forming]	−3.06	1.44E-04	29
P20767	ENSRNOG00000050000	Ig lambda-2 chain C region	−2.11	3.17E-06	7
P05544	Serpina3l	Serine protease inhibitor A3L	−2.04	3.67E-07	51
P20761	Igh-1a	Ig gamma-2B chain C region	−1.96	4.50E-06	24
P18589;Q499Q3	Mx2	Interferon-induced GTP-binding protein Mx2; Isoform of P18589, Interferon-induced GTP-binding protein Mx2	−1.93	1.71E-03	17
A0A0G2K477	ENSRNOG00000034190	Isoform of F1LPW0, Immunoglobulin heavy constant mu	−1.93	2.00E-06	16
A0A0G2K980;M0RBD5	ENSRNOG00000055375	Ig-like domain-containing protein; Isoform of A0A0G2K980, Ig-like domain-containing protein	−1.92	1.15E-05	2
A0A0G2JVP4;F1LM30;F1LPW0	ENSRNOG00000034190	Isoform of F1LPW0, Immunoglobulin heavy constant mu; Isoform of F1LPW0, Immunoglobulin heavy constant mu; Immunoglobulin heavy constant mu	−1.90	1.28E-07	23
D3ZF97	Erlec1	Endoplasmic reticulum lectin 1	−1.89	7.62E-04	4

Description of the top ten up- and downregulated proteins identified when comparing the proteomic profile of 12-week-old renal arteries from spontaneously hypertensive rats (SHR) and normotensive Wistar Kyoto rats (WKY). The number of unique peptides (precursors) identified per protein is included to support the identification.

We next investigated the presence of biological processes enriched in the protein list from renal artery samples using ClueGO (Fig. 4*E* and Table S7). We identified 15 clusters of related GO and KEGG terms, such as endoplasmic reticulum to Golgi vesicle-mediated transport, translation regulator activity, and nucleotide metabolic process (Fig. 4*E*).

**Figure 5:**
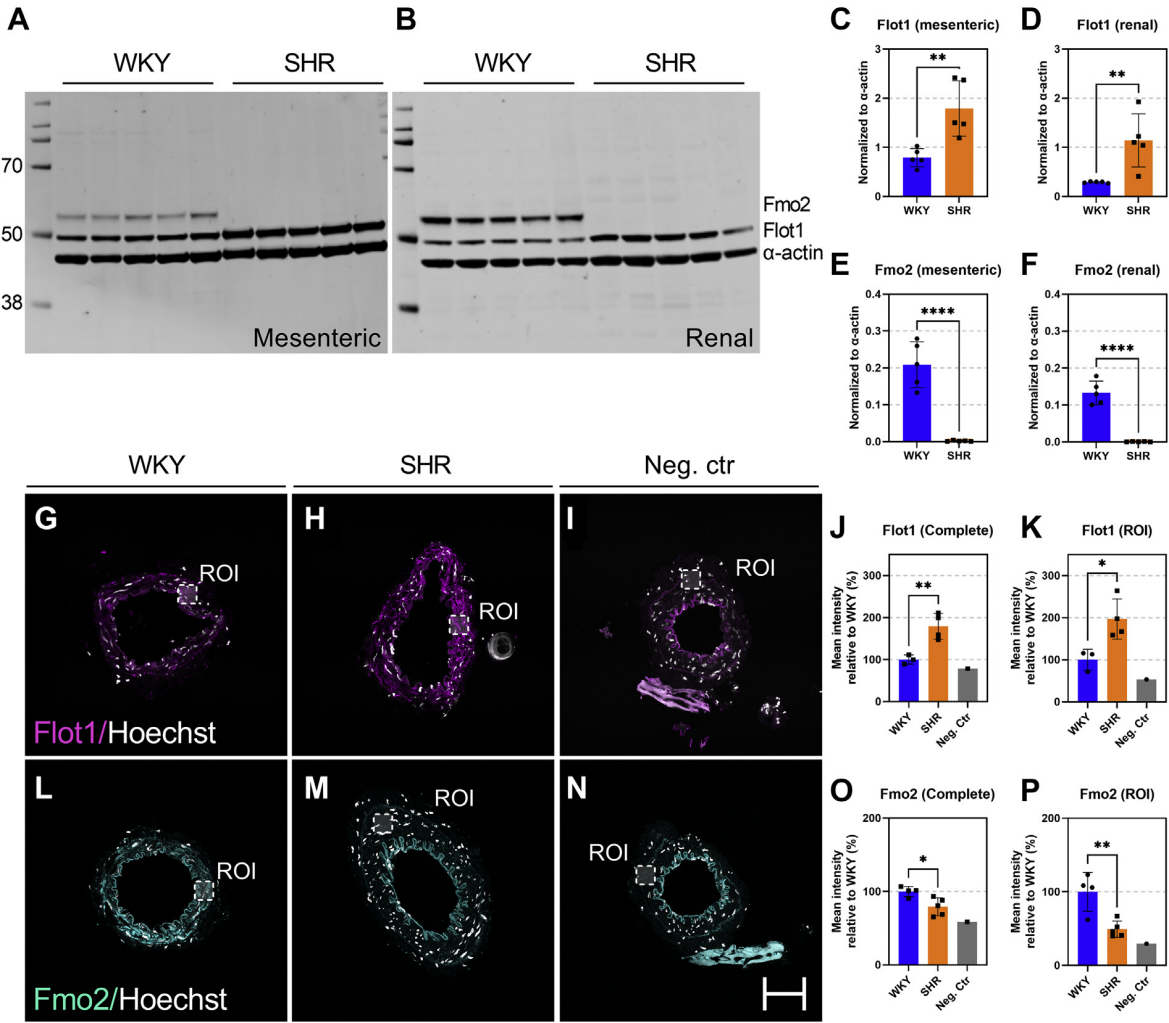
**Antibody-based validation of selected proteins.***A* and *B*, western blot analysis of flavin-containing monooxygenase 2 (Fmo2) and Flotillin-1 (Flot1) expression in mesenteric (*left*) and renal (*right*) arteries from Wistar Kyoto (WKY) control and spontaneously hypertensive rats (SHR), respectively. *C* and *D*, Flot1 expression normalized to α-actin in the two vascular beds. *E* and *F*, Fmo2 expression normalized to α-actin in the two vascular beds. N = 5 biological replicates in both western blots. Immunohistochemistry analysis of (Flot1, *magenta*, *G* and *H*) and flavin-containing monooxygenase 2 (Fmo2, *cyan*, *L* and *M*) in mesenteric arteries from WKY controls and SHRs (n = 2 biological replicates per condition, 2–3 technical replicates per rat). Cross sections used as negative control (Neg. Ctr) are shown in (*I*) and (*N*). Mean intensity measurement of Flot1 and Fmo2 in entire cross section (complete) or region of interest (ROI) is shown in (*J*), (*K*), (*O*), and (*P*), respectively. Scale bar = 50 μm. ∗*p* < 0.05, ∗∗*p* < 0.01, ∗∗∗∗*p* < 0.0001. Standard deviation is included on all column graphs.

To validate the MS data, we selected a protein from the top ten up- and down lists (Flotillin-1 (Flot1) and flavin-containing monooxygenase 2 (Fmo2)) that were regulated in both mesenteric and renal arteries and performed Western blot and immunohistochemistry (IHC) analysis. The density of bands for Flot1 were higher in SHRs compared with WKY control in both renal and mesenteric arteries (*p* = 0.006 and 0.008 in mesenteric and renal arteries, respectively; n = 5 in each group; Fig. 5, *A*–*D*). These data were supported by IHC analysis, which showed an increase in mean fluorescence intensity in mesenteric arteries from the SHR compared with WKY control (*p* = 0.009; Fig. 5, *G*–*K*). For Fmo2, no bands were detected in SHRs, while clear bands were detected in WKY controls (*p* ≤ 0.0001 in both artery types; n = 5 in each group; Fig. 5, *E* and *F*). This difference was supported in the IHC analysis where the Fmo2 mean fluorescent intensity was reduced in SHRs (*p* = 0.019; Fig. 5, *L*–*P*).

### Critical changes in protein expression across vascular beds

**Figure 6:**
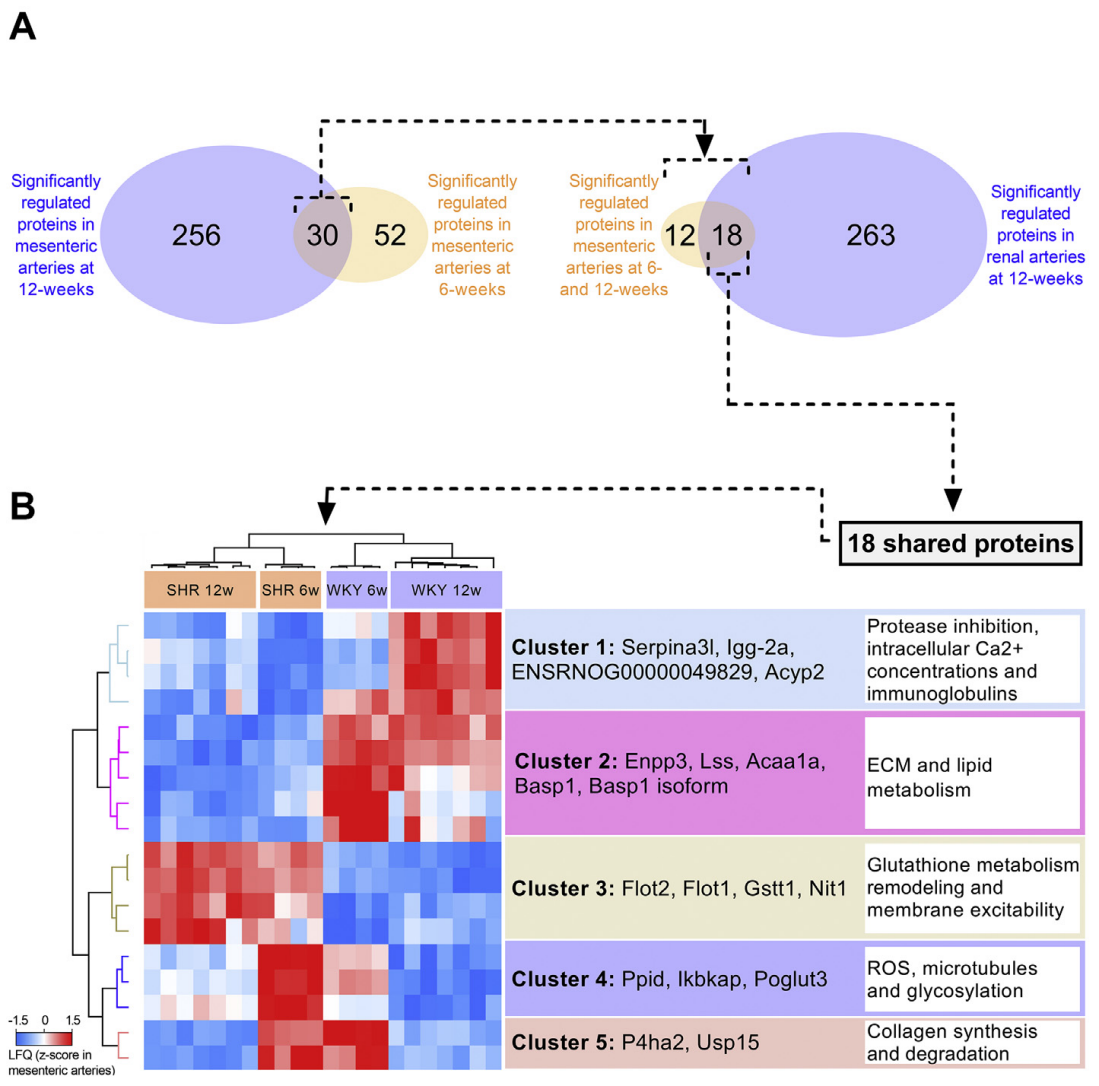
**Mapping of critical arterial changes occurring in the spontaneously hypertensive rat (SHR).***A*, Venn diagram representing number of significantly regulated proteins in previous Student *t* test analysis comparing 6- and 12-week-old mesenteric artery samples from the SHR and Wistar Kyoto (WKY) control. The number of shared proteins were subsequently compared with the significantly regulated proteins identified in renal arteries. *B*, unsupervised hierarchical clustering of 18 shared proteins identified in (*A*). Z-scored LFQ values from mesenteric arteries are depicted. Associated pathways were identified by literature mining. ECM, extracellular matrix; ROS, reactive oxygen species.

Our experimental setup enabled us to investigate the overlap of significantly regulated proteins between 6- and 12-week-old mesenteric arteries from the SHR compared with WKY control. We found that 30 proteins were shared between both stages (Fig. 6*A*), suggesting a central involvement in early BP regulation in the SHR. To clarify the potential importance of these in the vascular network in general, we compared the 30 proteins with the significantly regulated proteins identified in our renal artery analysis. This left us with 18 proteins (Fig. 6*A* and Table 4) that were changed across two different vascular beds and thus could contribute to a common regulatory mechanism of early-onset hypertension. Notably, the 18 proteins were identified after filtering for several criteria that limited false-positive discovery significantly, including: (1) more than two unique peptides per protein, (2) q-value (FDR) cutoff >0.05, significance cutoff by adjusted *p*-value >0.05, (3) removal of proteins that were potentially affected by selective inbreeding, (4) significance across two arterial beds. To elucidate the biological relevance, we performed an unsupervised hierarchical clustering analysis of the 18 proteins that revealed regulation of five clusters of proteins (Fig. 6*B*). Using literature mining on the clustered proteins, we identified associations to protease inhibition, intracellular Ca^2+^ concentration, immunoglobulins, ECM, lipid metabolism, glutathione metabolism, remodeling and membrane excitability, reactive oxygen species (ROS), microtubules, glycosylation, and collagen synthesis and degradation.

**Table 4:** Detected driver proteins

Protein group(s)	Genes	Protein description	Molecular weight	Unique peptides
A0A0A0MY07;Q9R085	Usp15	Isoform of Q9R085, Ubiquitin carboxyl-terminal hydrolase;Ubiquitin carboxyl-terminal hydrolase 15	109240.56;109254.59	18
A0A0G2JYL4;D3ZGT6	P4ha2	Isoform of D3ZGT6, Procollagen-proline 4-dioxygenase;Procollagen-proline 4-dioxygenase	60635.84;60867.06	28
A0A0G2K1L8	Basp1	Isoform of Q05175, Brain acid soluble protein 1	21719.25	2
D4A1G1;P35745	Acyp2	Isoform of P35745, Acylphosphatase;Acylphosphatase-2	13644.71;10863.3	6
F1LP76;Q8VHU4	Elp1	Isoform of Q8VHU4, Elongator complex protein 1;Elongator complex protein 1	149198.57;149170.64	22
F1LTN6	ENSRNOG00000049829	Uncharacterized protein	24914.85	20
P05544	Serpina3l	Serine protease inhibitor A3L	46277.17	46
P20760	Igg-2a	Ig gamma-2A chain C region	35185.86	25
P21775	Acaa1a	3-ketoacyl-CoA thiolase A, peroxisomal	43833.22	14
P48450	Lss	Lanosterol synthase	83300.54	16
P97675	Enpp3	Ectonucleotide pyrophosphatase/phosphodiesterase family member 3	99071.63	40
Q01579	Gstt1	Glutathione S-transferase theta-1	27468.29	12
Q05175	Basp1	Brain acid soluble protein 1	21790.27	20
Q566E5	Poglut3	Protein O-glucosyltransferase 3	58701.34	21
Q6DGG0	Ppid	Peptidyl-prolyl *cis*-*trans* isomerase D	40765.68	20
Q7TQ94;Q7TQ94-2	Nit1	Deaminated glutathione amidase;Isoform of Q7TQ94, Isoform 2 of Deaminated glutathione amidase	36093.54;36093.54-2	23
Q9Z1E1	Flot1	Flotillin-1	47499.4	54
Q9Z2S9	Flot2	Flotillin-2	47038.04	55

Description of the 18 proteins that are shared across two vascular beds (mesenteric and renal arteries) when comparing spontaneously hypertensive rats (SHRs) and normotensive Wistar Kyoto (WKY) rats. The number of unique peptides (precursors) identified per protein is based on the mesenteric artery analysis.

## Discussion

This study provides novel insight into proteins that are changed in small mesenteric resistance and renal arteries in SHR during the development of early-onset hypertension, most of which have never been associated with hypertension previously. Our experimental design allowed us to characterize protein changes before and after the development of hypertension in the SHR. In addition, by investigating protein changes in the renal arteries, we reveal 18 candidate proteins that change critically in different vascular beds. Only four out of the 18 proteins have been associated with hypertension (20, 21, 22, 23); however, the pathophysiological role of all proteins is undetermined. To achieve the comprehensive proteomic depth, we used a next-generation DIA-MS approach with a hybrid library combining HpH reversed-phase peptide fractionated DDA data and power of direct DIA. To our knowledge, we are the first to establish a rat mesenteric resistance artery-based library, which is ideal for a discovery-based proteomic investigation in rat arteries. The hybrid library has been made publicly available allowing others to access the data, from which new hypotheses can be generated, thereby advancing research in the hypertension field.

Several genetic differences exist between the SHR and WKY control strain because of selective inbreeding (24). Despite these differences, the WKY strain is considered the closest control available. To compensate for inbreeding differences, we included a comparison to an outbred Wistar Hannover strain. By using this additional strain, we could identify proteins that were likely to be due to genetic drift and changed protein expression in the WKY, rather than strain difference in BP, and remove these proteins from our analysis. Although not within the scope of this study, the number of significantly regulated proteins between the WKY and Hannover was surprisingly high (=1209), suggesting a high proteomic difference between the two control strains. This should be taken into consideration when groups compare conflicting data obtained from similar experiments in the different strains. Nevertheless, only 74 of the regulated proteins between the WKY and Hannover matched those in the SHR *versus* WKY list, which left 286 significantly regulated proteins that were associated with a strain difference in BP in the SHR mesenteric arteries.

This list represents the proteins that show the highest degree of expression changes from the SHR mesenteric arteries, which allowed us to investigate novel mechanistic pathways involved in the early stages of hypertension. Our enrichment analysis of the 286 regulated proteins revealed monocarboxylic acid metabolic process and negative regulation of hydrolase activity as the predominant pathways associated with early-onset hypertension. We observed that the monocarboxylic acid metabolic process cluster was particularly associated with lipid metabolism processes such as regulation of lipid metabolic process, fatty acid metabolic process, and regulation of lipase activity. The regulated proteins in this pathway suggest a change in the handling of lipids in the vascular wall of the SHRs. For instance, decreased APOA4, APOC1, and APOC3 expression would result in reduced lipid removal from the vascular wall (25) and inhibition of lipoprotein lipase activity (26), potentially promoting lipid accumulation, which might be associated with hypertension (27). Changes in the expression of proteins involved in signaling pathways can be difficult to interpret in a proteomic study such as this. The activation states of such proteins are not reflected by their overall level of expression, thus it can be difficult to determine whether the activity of a pathway is truly up- or downregulated. Therefore, it is important that future studies investigate the pathophysiological contribution of specific pathways detected in this study, which are potentially involved in the development of hypertension.

ECM proteins play a critical role in vascular stability and cell behavior, with dysfunction in this system linked to the pathogenesis of hypertension (6); however, a complete overview of the ECM-associated remodeling in hypertension has never been established. Thus, with the extensive proteomic coverage achieved in this study, we created a map of ECM changes in arteries from the SHR. Our data revealed 38 ECM-related proteins that were regulated in the SHR at 12 weeks of age, most of which have not been linked to hypertension previously. Interestingly, our data indicated that the quantified ECM proteins could be grouped into three distinct clusters based on (1) ECM glycoproteins, (2) ECM regulators, and (3) a mixture of ECM proteins. These clusters could distinguish the SHR from the WKY controls, supporting a phenotypical difference. Notably, some of the identified ECM glycoproteins in our study have been linked to vascular remodeling and stiffness previously. For example, elevated expression of MFGE8 was positively correlated with aortic stiffness in chronic kidney disease patients (28), and increased GAS6 levels in serum have been observed to correlate with elevated BP and age-related vascular remodeling in mice (29). In addition, increased plasma levels of SPARCL1 have been found in patients with maladaptive right ventricular function from pulmonary hypertension (30). We identified similar regulations of Mfge8, Gas6, and Sparcl1 in our study, supporting a pathological relevance of the identified ECM glycoproteins. The ECM regulators were highly abundant in 12-week-old WKY controls while the expression levels were lower in the three other groups. Several of these proteins, such as inter-alpha-trypsin inhibitor heavy chain (Itih2–4) and the serpins, improve ECM stability (31). Although their role in ECM stability in the vascular wall is yet to be determined, our data suggest that downregulation of these proteins in hypertension could destabilize the arterial ECM. Notably, the studies referred to here (28, 29, 30) investigated individual proteins and their pathological association to remodeling. While our MS method detected these proteins, our approach also identified many other coclustering proteins that were regulated and thus a more complex interplay between ECM proteins. This highlights the advantage of using a discovery-based DIA-MS approach to unravel the complex compositional and dynamic changes that occur during arterial wall remodeling.

Despite a large overlap of detected proteins between the mesenteric and renal arteries (=4135), the number of shared significantly regulated proteins was limited between the two artery types (=53). Other than these being different vascular beds, the renal arteries were also conduit arteries compared with resistance mesenteric arteries, which is likely to contribute to this proteomic difference. Despite the small overlap, one biological process (cellular modified amino acid metabolic process) was shared between the two pathway analyses of renal and mesenteric arteries. When comparing the associated proteins from both analyses, we observed that the majority (Eef1g, Idh1, Gsto1, Gstt1, and Gstt3) were associated with glutathione metabolism, a child term of the cellular modified amino acid metabolic process. It has been proposed that glutathione can serve as an intracellular thiol-disulfide redox buffer that can protect against oxidative stress due to its oxidizability (32). Increased levels of red blood cells containing glutathione in its oxidized state have been reported in untreated hypertensive patients (33). Furthermore, increased levels of superoxide O_2_^−^ have been observed in spontaneously hypertensive stroke-prone rats (34), linking glutathione metabolism to hypertension. The glutathione S-transferases (GSTs), such as Gsto1, Gstt1, and Gstt3, catalyze glutathione-dependent reactions leading to conjugation and detoxification of ROS in vascular smooth muscle cells (VSMCs) (35). As such, upregulation of Gsto1, Gstt1, and Gstt3 in the SHR could occur as a counteracting mechanism to cope with increased levels of vascular oxidative stress. Interestingly, another variant of GSTs (Gstm5) was observed to be reduced in renal specimens from hypertensive patients using genome-wide microarray expression profiling (36). We similarly observed a reduction of Gstm5 in mesenteric and renal arteries (*p* = 0.003 and 0.669, respectively). Although this supports our findings, it also implicates an inverse relationship between the expression levels of GST variants in the SHR (*e.g.*, upregulation of Gstt1/3 variants and downregulation of Gstm5).

Importantly, our experimental setup allowed us to reveal 18 proteins that were regulated significantly in pre- and early-onset hypertension in the mesenteric artery, which were also altered in the renal artery. From these 18 proteins, we identified five pathway clusters, which are likely to be driving the hypertensive phenotype in different vascular beds. Cluster 1 included four proteins Serpina3l, Igg-2a, ENSRNOG00000049829, and Acyp2 that associated with regulation of protease inhibition, intracellular Ca^2+^ concentrations, and immunoglobulins. Serpina3l is a serine protease inhibitor that also was included in the pathway analysis of 12-week-old mesenteric arteries (*negative regulation of hydrolase activity*; alias LOC299282). The inhibitor was among the most downregulated proteins in both artery types in the SHRs. These protease inhibitors might have a protective role against ECM remodeling, which is likely to be lost when downregulated. Interestingly, the SR calcium pump regulator Acyp2 enhances SERCA2a activity (37, 38), thereby regulating transport of cytosolic Ca^2+^ into the SR. The concentration of cytosolic Ca^2+^ in VSMCs regulates vascular tone and remodeling, which is critically associated with hypertension (39, 40, 41, 42). The expression level of Acyp2 was downregulated in 6- and 12-week-old SHRs, which could lead to changes in Ca^2+^ homeostasis. Interestingly, our data showed increased expression levels of Ryr2 in mesenteric arteries from the SHRs. The Ryr2 channel is also involved in releasing Ca^2+^ from the SR, supporting changes in intracellular Ca^2+^ regulation between the SHRs and WKY controls. These data suggest that a suppression of SERCA2a and increased level of Ryr2 reduce the Ca^2+^ levels in SR, increase cytosolic Ca^2+^, thereby affecting the contractile state of VSMCs.

Cluster 2 included Enpp3, Lss, Acaa1a, and Basp1, which associated with ECM and lipid metabolism. Enpp3 is a hydrolyzing glycoprotein involved in regulating extracellular nucleotides. Enpp3 mRNA expression was downregulated in VSMCs when exposed to angiotensin II (43). Although angiotensin II is not the main driver of increased BP in the SHR (44), the model has increased renin and angiotensin levels in serum (45), suggesting pathological similarities to the angiotensin II-induced model. Our data showed reduced Enpp3 expression in SHRs compared with WKY controls, which is likely due to increased angiotensin II levels in the SHR. Lss and Acaa1a are associated with lipid metabolism by regulating cholesterol and fatty acid synthesis. There is a strong link between dyslipidemia and hypertension, supporting the regulation of these proteins (46).

Cluster 3 included Gstt1, Nit1, Flot1, and Flot2 and associated with glutathione metabolism, remodeling, and membrane excitability. Both Gstt1 and Nit1 are linked to glutathione metabolism and were upregulated in both 6- and 12-week-old SHRs, supporting the glutathione metabolism pathway association that was observed in renal arteries. The flotillins, Flot1 and Flot2, are membrane-associated proteins that are involved in cell-matrix adhesion, endocytosis, and can assemble lipid rafts or microdomains that function as signaling platforms (47, 48). Our data showed that Flot1 and Flot2 were among the most upregulated proteins in SHRs, and we validated Flot1 changes by Western blot and IHC analysis, which supported the detection. Notably, expression levels of Flotillin-2 were significantly increased in cardiac intercalated disk fractions from both Dilated Cardiomyopathy (DCM) and Arrhythmogenic Right Ventricular Cardiomyopathy (ARVC) patients (49). Both diseases are associated with cardiomyocyte remodeling, and links increased flotillin-2 to a remodeling phenotype. Furthermore, decreased expression of the cardiac sodium channel Nav1.5 accompanied with impaired cardiac conductance was reported in Flotillin-1/2 knockdown mice compared with control (50). Flotillins have, to our knowledge, not been studied in relation to hypertension and vascular remodeling, but our study suggests a critical role of flotillins in the pathophysiology of hypertension.

Cluster 4 contained three proteins including Ppid, Ikbkap, and Poglut3 that associated with changes in ROS, microtubules, and glycosylation. Ppip or Cyclophilin D regulates the mitochondrial permeability transition pore and is a regulator of mitochondrial ROS generation (51). Angiotensin II-induced hypertension increased Ppip-associated ROS production in mice (52). Conversely, angiotensin II-induced hypertension was attenuated when using Ppip-depleted mice, demonstrating a link between ROS production and Ppip in the hypertensive mice (52). Our data also showed an increased expression of Ppip in SHRs, which is likely due to elevated angiotensin levels in the SHRs. Ikbkap (or Elp1) is a scaffold protein that can promote α-tubulin acetylation, thereby regulating microtubule network remodeling and dynamics (53, 54). We have previously shown that the microtubule network is an important trafficking pathway for certain proteins (55, 56). Although, little is known about the effect of hypertension on the microtubule network, one study reported that angiotensin II-treatment enhanced deacetylation and disassembly of tubulin in endothelial cells (57), questioning whether exposure to a high BP causes changes to the microtubule stability. Our data showed increased Ikbkap expression in SHRs compared with controls and could support a counteracting mechanism by Ikbkap in the SHRs. The Poglut3 enzyme facilitates *O*-glycosylation on epidermal growth factor (EGF)-like repeats on a variety of proteins during secretion (58). We found that Poglut3 expression increased in SHRs compared with WKY control. This is in alignment with our observation of several ECM-associated glycoproteins that increased in SHRs. However, changes in Poglut3 expression can affect multiple pathways, and therefore Poglut3 might contribute to the pathophysiology of hypertension in several ways. For example, glycosylation is also required for Notch processing and trafficking (58), and changes in Notch3 signaling have been proposed to play a critical role in VSMC differentiation and the pathogenesis of pulmonary arterial hypertension (59).

Cluster 5 included two proteins P4ha2 and Usp15 that associated with collagen synthesis and degradation. P4ha2 can facilitate the triple helix formation of collagen and regulates the hypoxia-inducible transcription factor HIFα (HIF-1α) by catalyzing 4-hydroxyproline residues (60). HIF-1α can modulate ECM formation *via* P4ha2 or other regulators such as Sox9 (61). We found decreased P4ha2 expression in SHRs that is in alignment with our matrisome profiling data, which also showed decreased expression of collagens, such as Col3a1 and Col5a3 in the SHRs compared with WKY control. The enzyme Usp15 catalyzes deubiquitination and regulates the ubiquitin-proteasome system by stabilizing monomers of ubiquitin on proteins. Inhibition of USP15 decreased collagen expression, including Col3a1, in TGF-β-stimulated fibroblast cell cultures, suggesting that USP15 can regulate collagen expression (62). Notably, our data showed decreased expression of both Usp15 and Col3a1 in SHRs, and the regulatory effect of Usp15 might therefore explain the downregulation of Col3a1.

Taken together, we identified associations to protease inhibition, intracellular Ca^2+^ concentrations, immunoglobulins, ECM, lipid metabolism, glutathione metabolism, remodeling and membrane excitability, ROS, microtubules, glycosylation, and collagen synthesis and degradation. Although elements of these pathways have been linked to hypertension previously (4, 63), this study is the first to show collective proteins that are linked with each pathway, which are dysregulated in the prehypertensive stage of the disease and not elicited by pressure changes. This study presents vast amounts of data implicating several novel proteins in the pathogenesis of hypertension, as well as highlighting these 18 proteins and their associate pathways to provide novel insight into the disease, which, with further investigation, have the potential to the become innovative therapeutic targets.

This is an explorative study, which is not hypothesis-driven. As such, we have not been able to investigate the physiological impact of the novel proteins we have identified in the pathogenesis of hypertension. However, the enrichment analysis provides mechanistic insights into the pathways involved in the development of hypertension, thereby creating a plethora of new hypotheses, which will be tested in future studies and substantially advance the field. Furthermore, our proteomic analysis did not differentiate between cell types found in the vascular wall. Although it would be advantageous to have cell-type specific changes in protein expression, the process of isolating cells can influence the protein expression profiles and limits the ability to analyze changes in ECM-associated proteins. As such, we see it as an advantage that the study is based on intact, freshly isolated arteries. Our proteomic analysis provides a firm foundation for hypotheses on cell-specific modifications of the vascular wall in hypertension.

In summary, this study has unraveled the deep proteomic complexity of mesenteric resistance arteries in SHRs and WKY controls. We identified changes in several ECM proteins providing novel insight into the vascular remodeling process observed in the SHRs. Additionally, our data reveal 18 proteins driving the prehypertensive state as well as early-onset hypertension. Our pathway analysis of these driver proteins demonstrates an involvement of multiple novel proteins and pathways that have not been associated with hypertension previously. Together, these data will generate new hypotheses and advance the field of hypertension.

## Experimental procedures

### Experimental animals

The animal experiments were approved by local Animal Care and Use Committees (institutional approval numbers P20-457 and P21-117). Experiments were performed in accordance with the directives of the Danish National Committee on Animal Research Ethics, and Danish legislation on experimental animals. In accordance with the methods of killing animals described in annex IV of the EU Directive 2010/63EU, rats were made unconscious by a single, percussive blow to the head. Immediately after the onset of unconsciousness, cervical dislocation euthanized the rats. Three cohorts of male SHRs (SHR/KyoRj) (14), WKYs (WKY/KyoRj), or Hannover rats (Janvier) at 6 weeks, 12 weeks (SHRs and WKYs), and 13 weeks (Hannover) of age were group housed and supplied with *ad libitum* water and food access (n = 4 (6-week groups), 7 (12-week groups), and 6 (Hannover group), respectively). Clean cages were provided once a week, and rats were kept on a 12 h/12 h light/dark cycle.

### Measurement of blood pressure

To avoid confounding effects of anesthesia in the proteomic analysis, three rats from each group were sampled in order to determine the BP. These rats were not included in the proteomic analysis, but represent the BP of the population of rats used in the study. Increased mean BP over a stable 5 min period was confirmed in SHRs compared with WKY (Mean BP ± standard deviation: SHR = 140.975 ± 29.468, WKY = 79.577 ± 13.393, n = 3 in each group). The BP was measured as described previously (64). In brief, rats were anesthetized with 5% isoflurane (35% oxygen and 65% nitrogen), intubated, and connected to a respirator (≈65 breaths/min; tidal volume 8 ml/kg). The left carotid artery was cannulated with a catheter connected to a pressure transducer (Statham P23-dB) for continuous monitoring of the BP. A heating plate was used to maintain the body temperature of the rats at 37 °C. After the experimental protocol, the rats were euthanized using cervical dislocation.

### Dissection of arteries

After the rats were euthanized, the intestines containing the mesenteric vascular bed and the main branch of the renal artery connecting the thoracic aorta to the kidney were excised and incubated in ice-cold physiological salt solution (PSS: 120 mM NaCl, 2.8 mM KCl, 1.5 mM CaCl_2_, 25 mM NaHCO_3_, 1.18 mM KH_2_PO_4_, 2.5 mM MgSO_4_, 0.03 mM EDTA, 5.6 mM D-glucose). Small mesenteric and renal arteries were dissected, collected in 1.5 ml Lobind centrifuge tubes (Eppendorf), snap frozen in liquid nitrogen, and stored at −80 °C. Small sections of mesenteric resistance artery (0.5–1 cm in length) were embedded in Tissue-Tek OCT (Sakura) for sectioning and staining.

### Protein isolation and quantification

Snap-frozen arteries were homogenized in 200 μl of ice-cold lysis buffer (50 mM Tris pH 8.5, 5 mM EDTA pH 8.0, 150 mM NaCl, 10 mM KCl, 1% NP-40 and 1× complete protease inhibitor cocktail (Roche)) by three rounds of chopping the tissue using dissection scissors and a handheld homogenizer. Homogenates were centrifuged at 11,000*g* for 10 min at 4 °C to obtain the supernatant. Protein quantification of the tissue extracts was determined by bicinchoninic acid assay (BCA) (Thermo Scientific).

### Tissue sectioning, staining, and imaging

Small mesenteric resistance arteries were sectioned in a cryostat microtome (Leica CM3050 S). Sections were cut at 10 μm thickness and attached to Superfrost Plus glass slides (VWR) and stored at −80 °C. Tissue sections for bright field imaging were stained with a Sirius red staining protocol. In brief, tissue sections were adjusted to room temperature (RT) and fixed in Bouin’s solution (Sigma) overnight (O/N). Sections were rinsed in Milli-Q water for 20 min, stained in Weigert’s solution for 10 min (filtered Weigert HTX A solution and Weigert HTX B-solution, Histolab), and rinsed in Milli-Q water for 5 min. Sections were stained in filtered Picro-sirius red solution for 15 min (Histolab), dehydrated in 99% EtOH, washed in xylen (Sigma), and allowed to air-dry before mounting in pertex (Histolab).

Tissue sections for fluorescent imaging were fixed in 4% PFA/1× PBS (15 min), washed in 1× PBS, blocked in blocking buffer (5% normal swine serum (Jackson ImmunResearch), 1% bovine serum albumin (BSA, Sigma), 0.1% TritonX-100 (Sigma) in 1× PBS), and stained with commercial anti-FLOT1 (HPA001393, 1:500) and anti-FMO2 (HPA028261, 1:300) from (Sigma) diluted in 1% BSA, 0.1% TritonX-100 (Sigma) in 1× PBS O/N at 4 °C. Sections were counterstained with anti-rabbit secondary antibodies cross-absorbed to Alexa Flour 555 (1:400) diluted in 1% BSA, 0.1% TritonX-100 in 1× PBS for 1 h at RT. Washes in a washing buffer (0.25% BSA, 0.1% TritonX-100 in 1× PBS) were used between and after antibody staining. Hoechst 33342 (1:1000, Invitrogen) was added to secondary antibody staining. Sections were mounted in anti-fade mounting medium (ProLong Diamond Antifade Mountant, Invitrogen).

Bright field images were acquired on a Zeiss Axio Scan.Z1 slide scanner using a 20×/0.8 Plan-Apochromat objective lens (Zeiss). Images were cropped to individual arterial cross sections and analyzed, blinded, in ZEN (v3.2, blue edition) software. A profile ruler tool was applied to measure the media and lumen diameters (minimum diameter) of each cross section, and the ratio was calculated. Fluorescent images were acquired on an upright laser scanning confocal microscope using a 63×/1.4 Oil Plan-Apochromat objective lens (Zeiss). A tile scan (2 × 2 tiles) was used to ensure imaging of the entire arterial cross section. Mean intensity measurements were acquired in ImageJ (Fiji) (v2.1.0/1.53f/Java 1.8.0_172) by measuring the entire cross section and a region of interest (ROI; 20 × 20 μm). Measurements were converted to percentage relative to WKY control. Statistical analysis was performed in GraphPad Prism (v9) using unpaired Student *t* test.

### Western blot analysis

Tissue extracts were dissolved in SDS-sample buffer (NuPAGE LDS Sample Buffer (4×), Thermo Scientific) containing 0.1 M DTT (NuPAGE Sample Reducing Agent (10×), Thermo Scientific) and heat-treated for 10 min at 70 °C. Proteins were separated by gel-electrophoresis on 4 to 12% Bis-Tris SDS-PAGE gels (Invitrogen) and transferred onto polyvinylidine difluoride (PVDF) nitrocellulose membranes (Immobilon-FL, Millipore). The membranes were blocked in Odyssey Blocking buffer (Li-Cor Biosciences) and incubated with primary antibodies anti-Flot1 (HPA001393, 1:1000, Sigma), anti-Fmo2 (HPA028261, 1:1000, Sigma) or anti-alpha smooth muscle cell actin (ab32575, 1:2500, Abcam) O/N at 4 °C. The membranes were washed (PBS-Tween 0.1%) and incubated with conjugated secondary antibodies (α-Rabbit 680 or 800, Li-Cor Biosciences; 1:10,000, respectively) for 1 h at RT. Proteins were visualized using an Odyssey Infrared Imaging System (Li-Cor Biosciences) and analyzed with supplier’s software (Image Studio Lite, v5.2.5). Protein bands corresponding to Flot1 and Fmo2 were normalized to their respective α-actin band, and intensities from SHR and WKY controls were compared. Statistical analysis was performed in GraphPad Prism (v9) using unpaired Student *t* test.

### Sample preparation for proteomic analysis

Tissue extracts (100 μg) were diluted in digestion buffer (0.5% SDC in 50 mM TEAB), heat-treated for 5 min at 95 °C, and prepared by a modified filter-aided sample preparation (FASP) protocol (65). In brief, tissue extracts were transferred to 0.5 ml (tilted) spin filters (Amicon), centrifuged at 14,000*g* for 15 min, and reduced and alkylated by addition of digestion buffer containing 1:50 (v:v) tris(2-carboxyethyl)phosphine (0.5 M, Sigma) and 1:10 (v:v) 2-chloroacetamide (Sigma) for 30 min at 37 °C. Samples were digested in fresh digestion buffer containing 1 μg Typsin/LysC mix (Promega) and 0.01% ProteaseMAX (Promega) O/N at 37 °C. Digested samples were desalted using stage-tips containing styrene divinylbenzene reversed-phase sulfonate material (SDB-RPS; 3 M).

The mesenteric artery-based library was generated using a pooled digested and stage-tipped sample from the 12-week-old SHRs and WKYs that was fractionated. A high-pH reverse-phase peptide (HpH) fractionation kit (Pierce, Thermo Scientific) was used to create the 15 fractionations.

### Data acquisition by liquid chromatography–mass spectrometry (LC-MS)

Peptides were separated on 50 cm columns packed with ReproSil-Pur C18-AQ 1.9 μm resin (Dr Maisch GmbH). Liquid chromatography was performed on an EASY-nLC 1200 ultra-high-pressure system coupled through a nanoelectrospray source to an Exploris 480 mass spectrometer (Thermo Fisher Scientific). Peptides were loaded in buffer A (0.1% formic acid) and separated applying a nonlinear gradient of 5 to 65% buffer B (0.1% formic acid, 80% acetonitrile) at a flow rate of 300 nl/min over 100 min. Spray voltage was set to 2400 V. Data acquisition switched between a full scan (120,000 resolution, 45 ms max. injection time, AGC target 300%) and 49 DIA scans with isolation width of 13.7 m/z and windows overlap of 1 m/z spanning a precursor mass range of 361 to 1033 m/z (15,000 resolution, 22 ms max. injection time, AGC target 1000%). Normalized collision energy was set to 27.

### Protein identification by computational data analysis

Raw DDA and DIA files were first searched in Spectronaut (14.6.201001.47784) using the Pulsar search engine to generate the hybrid library. Identification settings: Digest type = specific, missed cleavage = 2, min peptide length = 7, max peptide length = 52, digestion rule = Trypsin/P; Identification settings: Peptide, protein and PSM FDR = 0.01; Spectral library filters = m/z 1800 to 300, Precursor min and max = 6 and 3, Best N fragments per peptide = True, missed cleavage = false, modification = none. Tolerance settings: Searches were set to Dynamic and Factor was set to 1. The generated hybrid library was used for library-based DIA analysis using default settings. Data were searched against UniProt FASTA database (UP000002494_10116.fasta (21,587 entries) and UP000002494_10116_additional.fasta (9981 entries), August 2020). Label-free quantification was performed in Spectronaut using default manufacturer settings.

### Bioinformatic analysis of MS data

All downstream data analysis was performed in Perseus (v1.6.14.0) (66) and R (v4.0.3). Protein groups from the datasets were filtered by ≥2 unique peptides (precursors identified in Spectronaut) and minimum 75% valid values in each group. Data were log2 transformed and missing values were imputed (width = 0.2, down shift = 1.8). Volcano plots and two-sided Student *t* test were generated using 250 randomizations, permutated FDR <0.05, and *p* < 0.05. ECM enrichment was achieved by comparing with a curated matrisome gene list (18, 19) and selecting overlapping proteins for further analysis. Hierarchical clustering was based on z-scored LFQ values and generated by average linkage, preprocessing with k-means, and Euclidean distance. The z-score normalization was calculated by subtracting mean intensity from each protein value across all samples followed by division by the standard deviation. ClueGO network analysis was performed in Cytoscape (67) (v3.8.1) using the ClueGo app (17) (v2.5.7). In brief, *Rattus norvegicus* was selected as organism, significantly regulated proteins were added, and the Gene Ontology (GO) biological processes (GO-BiologicalProcess, CellularComponent, ImmuneSystemProcess, MolecularFunction-EBI-UniProt-GOA-ACAP-ARAP, downloaded 15.01.2021) and Kyoto Encyclopedia of Genes and Genomes (KEGG, downloaded 15.01.2021) with the kappa-score = 0.4 were used. Two-sided hypergeometric test was used with false-discovery rate (FDR) corrected for multiple testing (Bonferroni step down, *p* ≤ 0.05) and GO term fusion was enabled. A minimum of three genes and 4% genes per term were applied.

## Data availability

MS raw files and hybrid libraries have been deposited to the ProteomeXchange Consortium *via* PRIDE (68) with the identifier PXD026051.

## Supporting information

This article contains supporting information.

## Conflict of interest

The authors declare that they have no conflicts of interest with the contents of this article.
