# Supplementary material for: Identification of novel proteins and mechanistic pathways associated with early-onset hypertension by deep proteomic mapping of resistance arteries
Source: J Biol Chem. 2021 Dec 18;298(1):101512. doi: 10.1016/j.jbc.2021.101512 (PMC8760518; doi:10.1016/j.jbc.2021.101512)

**Supplementary Figure 1: Spectral library and number of identifications**. Usage of hybrid DIA library resulted in identification of most protein groups, peptides and precursors in both mesenteric and renal arteries (mA and rA, respectively). HpH-fractionation = high-pH-fractionation.


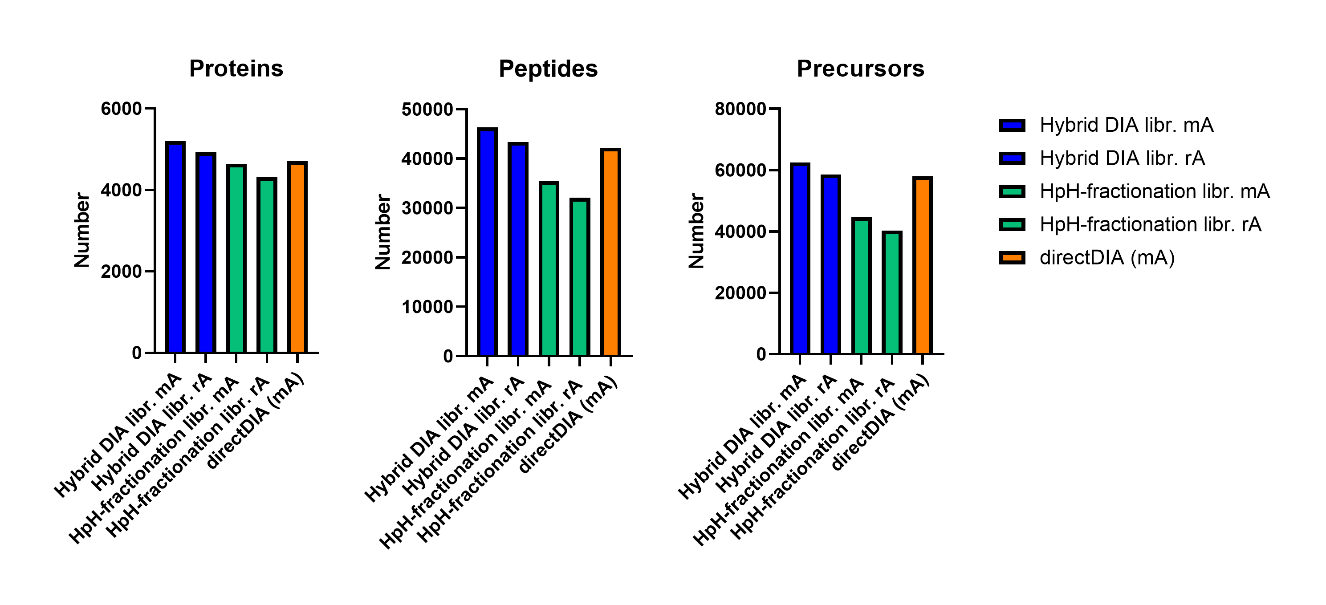

Supplement: Supplemental Figure S1 [file mmc8.docx]
